# The Bacterial Profile and Antimicrobial Susceptibility Patterns of Urinary Tract Infection Patients at Pawe General Hospital, Northwest Ethiopia

**DOI:** 10.1155/2022/3085950

**Published:** 2022-04-25

**Authors:** Abayeneh Girma, Aleka Aemiro

**Affiliations:** Department of Biology, College of Natural and Computational Science, Mekdela Amba University, P.O. Box 32, Tuluawlia, Ethiopia

## Abstract

Urinary tract infection remains the most common infection widespread worldwide in both community and hospital settings. Rapidly increasing antibiotic resistance of uropathogens is resulting in limited treatment options. Thus, understanding the current uropathogens and their antimicrobial susceptibilities is essential for effective urinary tract infection treatment. The purpose of this study was to isolate, characterize, and determine the antimicrobial susceptibility patterns of bacterial pathogens associated with urinary tract infection at Pawe General Hospital in Northwest Ethiopia. A hospital-based cross-sectional study design was conducted from January to April, 2020, at Pawe General Hospital. Midstream urine specimens were collected from 141 individuals with suspected urinary tract infection for bacteriological identification and antimicrobial susceptibility testing. Among the 141 study participants, twenty-nine (20.6%) showed significant bacteriuria. *Escherichia coli* (42.6%) had the highest proportion of isolated uropathogen followed by *Klebsiella* spp. and *Pseudomonas* spp. each (10.7%); *Proteus* spp. (9.3%); coagulase negative staphylococci, *Staphylococcus aureus*, and *Enterobacter* spp. each (6.7%); *Citrobacter* spp. (4%); and *Enterococcus faecalis* and *Streptococcus* spp. each (1.3%). Outpatient isolates showed a resistance of 64% and 78.6% to amoxicillin-clavulanic acid and tetracycline, respectively. Inpatients showed 63.9% and 87.2% of resistance to cephalexin and tetracycline. It was also observed that all the isolates have a multiple antimicrobial resistance index greater than 0.20 except *Citrobacter* spp. (0.142) in inpatients. Even though in this locality, most isolates were sensitive to ceftriaxone, gentamicin, ciprofloxacin, nitrofurantoin, and norfloxacin, they are considered appropriate antimicrobials for empirical treatment of urinary tract bacterial infections. Periodic monitoring of etiology and drug susceptibility is highly recommended, along with health education on the transmission and causes of urinary tract infection.

## 1. Introduction

Urinary tract infections (UTIs) are infections caused by infectious agents (bacteria, fungi, virus, and parasites) present and propagate in any part (bladder = cystitis, kidney = pyelonephritis, urethra = urethritis, ureters = urethritis, and urine = bacteriuria) of the urinary tract [1]. The infection of the urinary tract can either be asymptomatic or symptomatic and can occur in uncomplicated (UTIs that occur in a normal genitourinary tract with no prior instrumentation) or complicated (infections that diagnosed in genitourinary tracts that have structural or functional abnormalities, including instrumentation such as indwelling urethral catheters, and are frequently asymptomatic) individuals [2]. Annually, one hundred fifty million people are diagnosed with urinary tract infections across the world and subsequently spend around six billion dollars on health care [1, 2]. In low-income countries like Ethiopia, UTIs lead to treatment failures and long-term hospital complications, threaten our ability to perform modern medical procedures, impose a major economic burden on society, and finally result in too much morbidity and mortality [1–3].

Compared to other uropathogens, bacterial urinary tract infections are the most common and dangerous infections in humans and occur frequently in the community and hospital environments [1–4]. *Escherichia coli*, *Klebsiella* spp. *Enterobacter* spp., *Proteus* spp., *Pseudomonas* spp., *Acinetobacter* spp., *Serratia* spp., and *Citrobacter* spp. are Gram-negative bacteria with the most causative agents covering ninety percent of UTIs. Group B *streptococci*, *Enterococcus* spp., and *Staphylococcus* spp. are from the Gram-positive bacteria responsible for the remaining ten percent of UTI cases [3, 4]. Of all, *E. coli* are the most common and frequently isolated uropathogens accounting for sixty-five up to ninety percent of the urinary tract bacterial infections [1–6]. Depending on the different factors like age, sex, catheterization, hospitalization, and previous exposure to antimicrobials; the relative frequency of both Gram-positive and Gram-negative bacterial pathogens may greatly vary.

Treatment and therapy for urinary tract infections is determined experimentally by the antimicrobial susceptibility testing. In Africa, however, it is observed that due to the high level of poverty, illiteracy, and poor hygienic practices, the easy availability and low cost of drugs, the high prevalence of fake and spurious drugs, the uncontrolled prescription and usage of antibiotics, and the lack of rapid laboratory facility for sensitivity test contributes to the emergence of resistant bacterial infections especially in uropathogens. The resistant strains of bacteria can be accelerated and spread by the transfer of resistant genes among species and genera through horizontal gene transfer (transformation, transduction, or/and conjugation) with mobile genetic elements (plasmids, transposons, or/and bacteriophages). This process results in the development and dissemination of new antimicrobial resistance varieties and novel resistance mechanisms among uropathogens due to the presence of R-plasmids that mediate resistance genes against various classes of commonly used antimicrobial agents. Since antibiotic resistance rates of pathogenic bacteria may vary from country to country, regionally and locally, and can also change rapidly with time, as such, they need to be monitored and managed closely because of their public health implications and impacts [5, 6].

Regarding the resistance rates in Ethiopia, different reports showed that a high incidence of resistance to the commonly used antimicrobial agents was observed [1, 2, 4–6], even though there are few published information available concerning the etiology and resistance patterns of urinary tract bacterial infections in some hospitals of Ethiopia. To the best of our knowledge, there is no previous study and published information on UTIs in the study area. Thus, this study was aimed to assess bacteriological profile and antimicrobial susceptibility patterns of symptomatic urinary tract infection among patients in Pawe General Hospital (PGH).

## 2. Materials and Methods

### 2.1. Study Setting, Design, and Period

Hospital-based cross-sectional study was conducted at Pawe General Hospital from January to April 2020. Pawe General Hospital is the mere public hospital found in Pawe town and provides various health services for routine cases. The hospital was chosen because it covers both rural and urban areas of the town. The town is found at a distance of 573 km from Addis Ababa which is the capital city of Ethiopia.

### 2.2. Sample Size Determination

Single population proportion formula was used to determine the sample size.

(1) $$\begin{array}{c} n=\frac{Z^{2}P\left(1-P\right)}{d^{2}}, \end{array}$$

where, *Z* = *z-*score for 95% confidence interval = 1.96. *P* = prevalence, and *d* = tolerable error = 5% .

Since there was no data in Ethiopia, the prevalence of UTI among both inpatients and outpatients was taken from Chad (32.7%) which was done by Kengne et al. [7]

(2) $$\begin{array}{c} n=\frac{{\left(1.96\right)}^{2}\times 0.327\left(1-0.327\right)}{{\left(0.05\right)}^{2}}=338+10\%\text{contigency},128+13=141. \end{array}$$

Therefore, a total of 141 UTI inpatient and outpatients were included in the study from the hospital.

### 2.3. Inclusion and Exclusion Criteria

One hundred forty-one patients to whom a cytobacteriological examination of urine was prescribed and who had not received antimicrobials within the previous two weeks were eligible for inclusion due to the fact that the antibiotic must have inhibited or destroyed the pathogens. UTI patients who were not willing to participate were excluded from this study.

### 2.4. Sample Collection

Clean catch mid-stream urine sample collection approach with aseptic measures was applied to minimize contamination of the sample [8]. A total of 141 fresh midstream urine samples were collected from both inpatient and outpatient UTI suspected individuals using sterile screw-capped universal container. The specimens were appropriately labeled and immediately processed after sampling.

### 2.5. Culturing and Identification of Isolates

The collected urine samples were spread on cysteine lactose electrolyte-deficient medium, MacConkey agar, and blood agar (Oxoid, UK) using L-shaped glass spreader and incubated aerobically at 37°C for 24 h. A significant bacteriuria was considered if urine culture yields ≥10^5^ CFU/mL. All positive urine cultures with significant bacteriuria were further identified by their colony characteristics, Gram-staining reaction, and pattern of biochemical profiles using standard procedures. *Enterobacteriaceae* were identified by H_2_S production and carbohydrate utilization tests in TSI agar, motility test, urease test, and IMViC (indole, methyl red, Voges-Proskauer, and citrate utilization) tests. The Gram-positive bacteria were identified using catalase and coagulase tests [9].

### 2.6. Standardization of the Inoculum

0.5 McFarland standard was prepared by mixing 0.50 mL of a (1.175% w/v) dehydrate barium chloride (BaCl_2_.2H_2_O) solution with 99.50 mL of (1% v/v) sulfuric acid (H_2_SO_4_) with constant stirring in a graduated cylinder. The turbidity standard solution was aliquoted into test tubes identical to those used to prepare the inoculum suspension. In addition, the absorbance of the prepared 0.5 McFarland standard solution was further confirmed to be 0.08 to 0.12 at 625 nm using spectrophotometry. To prevent evaporation, the tube containing a standard solution was tightly sealed and stored at room temperature. Before comparing with the bacterial suspension, the turbidity standard tube was vigorously mixed by the vortex mixer which makes a uniform turbid appearance [10].

### 2.7. Inoculum Preparation

From an overnight culture, 3–5 morphologically identical bacterial colonies were suspended in 5 mL of normal saline (0.85%; 8.5g/L NaCl) vigorously mixed by the vortex mixer and comparing to that of 0.5 McFarland standards which are approximately equivalent to 1.5 × 10^8^ CFU/mL. After adjusting the turbidity, a sterile cotton swab was dipped into the suspension and streaked over the entire surface of the prepared medium by rotating the plate in 60^o^ to ensure the even distribution of the inoculum [10].

### 2.8. Antimicrobial Susceptibility Testing

Antimicrobial susceptibility testing was performed using Kirby-Bauer's disk diffusion method [11]. The antimicrobial agents were selected due to the physicians prescribed for treatment of UTIs in the study setting. The standard commercially available (Oxoid, UK) antibiotic discs, namely, amoxicillin/clavulanic acid (AMC, 20/10 *µg*), cephalexin (CEPH, 30 *µg*), ceftriaxone (CRO, 30 *µg*), tetracycline (TE, 30 *µg*), gentamicin (CN, 10 *µg*), ciprofloxacin (CIP, 5 *µg*) nitrofurantoin (F, 300 µg), and norfloxacin (NOR, 10 *µg*) were used. These discs were aseptically laid with proper spacing on the surface of the inoculated agar plates and then pressed firmly onto the agar with sterile forceps and incubated at 35–37°C for 18–24 hours. The diameter of inhibition around the discs was measured to the nearest millimeter and interpreted as sensitive (S), intermediate (I), or resistant (*R*) according to the annually published microbiological breakpoints of CLSI [12]. Bacterial isolates resistant to three or more antimicrobials belonging to the different structural classes were considered multidrug resistant (MDR).

### 2.9. Quality Control

All materials, equipment, and procedures required for the study were adequately controlled. All specimens were immediately processed after collection. Only specimens which produced ≥10^5^ CFU/mL of urine were considered significant for the study. *E. coli* (ATCC^®^ 25922), and *S. aureus* (ATCC^®^ 25923) standard reference strains were used for testing sterility and performance of culture media and antibiotic discs. Generally, CLSI 2020 guidelines were strictly followed.

### 2.10. MARI Determination

The multiple antimicrobial resistance index (MARI) is the ratio between the number of antibiotics resisted by the pathogen (*a*) and the total number of antibiotics used in this study (*b*).

### 2.11. Ethical Considerations

Ethical clearance was obtained from Pawe General Hospital Administration. A written informed consent was also obtained from each participant before collecting the data. All information obtained in the course of the study were kept confidential and used solely for the purpose of the study.

### 2.12. Data Analysis

Data were entered and analyzed using SPSS version 25.0 software. Discrete variables were expressed as frequencies and percentages. The results were presented by tables.

## 3. Results and Discussion

Urinary tract infections (UTIs) are one of the most common and serious infections of the human urinary system that occurred in the community and hospital environments. UTIs are most often treated empirically and the antimicrobial agent selection criteria are determined on the basis of the pathogen type and its expected antimicrobial resistance patterns. Also, the management of UTIs has been jeopardized by the increase in the incidence of antimicrobial drug resistance. Thus, there is a need for periodic monitoring of the pathogens of UTIs and their antimicrobial susceptibility pattern in the locality [13].

In the present study, the details of culture characterization are shown in Table 1. From 141 samples collected in inpatient and outpatient settings, 29/141 (20.6%) samples were positive urine cultures, from which 75 bacterial isolates were obtained. *E. coli* (42.6%) was the most predominant bacterium isolated from urine, followed by *Klebsiella* spp. and *Pseudomonas* spp. each (10.7%); *Proteus* spp. (9.3%); coagulase negative staphylococci (CNS), *S. aureus*, and *Enterobacter* spp. each (6.7%); *Citrobacter* spp. (4%); *E. faecalis and Streptococcus* spp. each (1.3%), and this study was similar to the previous studies of Kibret and Abera [13] and Alemu et al. [14]. However, the observed high proportion of *E*. *coli* was varied with previously published studies conducted in Nigeria by Ekwealor and his colleagues [15] where *Staphylococcus* spp. was found to be the predominant urinary tract pathogen. This might be due to the variation of UTI-causing bacterial pathogens, differences in sample size, specimen collection technique, and study settings.

In the current study, the frequency of inpatient 47/75 (62.7%) uropathogens was greater than outpatients 28/75 (37.3%) (Table 1). This is in line with the previous studies of Kengne et al. [7] that revealed bacteriuria was more present in inpatients (70.4%) compared to outpatients (29.6%). Tesfa et al. [16] also further confirmed that inpatients were two times more likely to have culture positive results than outpatients. This might be due to the hospitalization, comorbidity, long-term antibiotic treatment, and immunocompromised conditions. In this study, Gram-negative bacterial isolates were more prevalent (80%) than Gram-positives (20%). This finding is in agreement with studies done in Jimma, Jijiga, Dire Dawa, and Shashamane, where 80.9%, 76%, 73.1%, and 59.2% of the isolates were Gram-negatives [2, 17–19]. Differences in characterization and identification methods are known to influence the relative prevalence of bacteria, which makes comparison difficult. Bacterial etiologies of UTIs can also vary across regions and over time within a population.

Antimicrobial resistance is a major clinical problem in treating infections caused by different bacterial pathogens and has increased over time. In this study, antimicrobial susceptibility pattern of inpatients showed that most of the isolates were sensitive to nitrofurantoin (100%), ciprofloxacin (100%), norfloxacin (97.9%), gentamicin (89.4%), ceftriaxone (55.3%), cephalexin (36.1%), and tetracycline (12.8%). Among outpatients tested for the available drugs, the isolates respectively showed susceptibility of (100%), (96.4%), (67.9%), (53.6%), (36%), and (21.4%), to nitrofurantoin and ciprofloxacin, norfloxacin and gentamicin, ceftriaxone, cephalexin, amoxicillin-clavulanic acid, and tetracycline (Tables 2 and 3). Ceftriaxone, norfloxacin, gentamicin, ciprofloxacin, and nitrofurantoin showed greater than 55% activity against all the isolates. These could be an excellent choice for empirical therapy of UTI in the study setting. However, prescription of these antibiotics depends on the patient's health status [15, 20–23]. Comparatively, the majority of the isolated uropathogens were resistant to amoxicillin-clavulanic acid and tetracycline [13, 15, 20–24]. This high level of resistance observed with amoxicillin-clavulanic acid and tetracycline might be due to the self-medication and irrational drug utilization habits of the communities for treating all kinds of bacterial infections.

MARI is a tool that indicates the spread of antimicrobial resistance in a given study population. In this study, 0.142–0.714 and 0.250–0.625 were the inpatient and outpatient MARI values of the isolates, respectively (Tables 4 and 5). Only *Citrobacter* spp. (0.142) gave MARI of <0.20 while others gave higher MARI. Similarly, [15] reported that only *Streptococcus* spp. (0.125) was < 0.20 MARI value. Any MARI greater than 0.20 implies that the strains of such bacteria originate from an environment where several antibiotics are used or misused [25]. This implies that a very large proportion of the bacterial isolates have been exposed to several antibiotics and thus have developed resistance to these antibiotics. Similar incidence was reported in the work of Ekwealor et al. [15] and Oli et al. [25] though not exactly with the same set of antibiotics.

### 3.1. Limitations of the Study

The study did not include the patients' clinical data such as age, gender, and being catheterized and noncatheterized and other associated risk factors of the patients. Due to the COVID-19 pandemic, species level identification and the presence of genetic resistance determinants were not performed.

## 4. Conclusions

The overall prevalence of UTI in the study participants was 20.6% (29/141). In this study, *E. coli*, *Klebsiella* spp., *Pseudomonas* spp., and *Proteus* spp. were the most prevalent among the investigated uropathogens. Inpatients had a higher risk of developing bacterial infections compared to outpatients. Ceftriaxone, gentamicin, ciprofloxacin, nitrofurantoin, and norfloxacin are considered as appropriate antimicrobials for empirical treatment of UTIs in the study area. Greater than 0.2 MARI values of the uropathogens from urine samples of this study underscore the need for continuous monitoring of the etiology and antimicrobial susceptibility testing of bacteria, along with health education on the transmission and causes of urinary tract infection.

## Figures and Tables

**Table 1:** Distribution of uropathogens in patients.

Urinary tract bacterial isolates	Number of isolates (N)		Percentage (%)	
	Inpatient	Outpatient	Inpatient	Outpatient
*E. coli*	21	11	33.60	32.50
*Klebsiella* spp.	5	3	15.81	15.00
*Proteus* spp.	4	3	13.84	13.50
*Pseudomonas* spp.	5	3	9.88	11.00
CNS	3	2	7.91	9.00
*Staphylococcus aureus*	3	2	6.72	6.50
*Enterobacter* spp.	3	2	5.92	5.50
*Citrobacter* spp.	2	1	3.95	3.50
*Enterococcus faecalis*	1	0	2.37	2.00
*Streptococcus* spp.	0	1	0.0	1.50
Total	47	28	100%	100%

CNS : coagulase negative staphylococci.

**Table 2:** Antimicrobial susceptibility pattern of uropathogens isolated from urine specimens of inpatients.

Bacterial pattern	Isolates	Antimicrobial susceptibility pattern, *n* (%)							
		Amoxicillin/C	Cephalothin	Ceftriaxone	Tetracycline	Gentamicin	Ciprofloxacin	Nitrofurantoin	Norfloxacin
*E*. *coli* (*N* = 21)	S	—	5 (23.8)	8 (38.1)	1 (4.8)	19 (90.4)	21 (100)	21 (100)	21 (100)
	I	—	1 (4.8)	1 (4.8)	1 (4.8)	1 (4.8)	0 (0.0)	0 (0.0)	0 (0.0)
	R	—	15 (71.4)	12 (57.1)	19 (90.4)	1 (4.8)	0 (0.0)	0 (0.0)	0 (0.0)

*Klebsiella* spp. (*N* = 5)	S	—	2 (40)	3 (60)	0 (0.0)	4 (80)	5 (100)	5 (100)	5 (100)
	I	—	1 (20)	0 (0.0)	1 (20)	1 (20)	0 (0.0)	0 (0.0)	0 (0.0)
	R	—	2 (40)	2 (40)	4 (80)	0 (0.0)	0 (0.0)	0 (0.0)	0 (0.0)

*Proteus* spp. (*N* = 4)	S	—	2 (50)	2 (50)	0 (0.0)	3 (75)	4 (100)	4 (100)	4 (100)
	I	—	0 (0.0)	1 (25)	1 (25)	0 (0.0)	0 (0.0)	0 (0.0)	0 (0.0)
	R	—	2 (50)	1 (25)	3 (75)	1 (25)	0 (0.0)	0 (0.0)	0 (0.0)

*Pseudomonas* spp. (*N* = 5)	S	—	0 (0.0)	1 (20)	0 (0.0)	3 (60)	4 (80)	3 (60)	3 (60)
	I	—	1 (20)	0 (0.0)	0 (0.0)	0 (0.0)	1 (20)	2 (40)	1 (20)
	R	—	4 (80)	4 (80)	5 (100)	2 (40)	0 (0.0)	0 (0.0)	1 (20)

CNS (*N* = 3)	S	—	1 (33.3)	2 (66.7)	0 (0.0)	3 (100)	3 (100)	3 (100)	3 (100)
	I	—	0 (0.0)	0 (0.0)	0 (0.0)	0 (0.0)	0 (0.0)	0 (0.0)	0 (0.0)
	R	—	2 (66.7)	1 (33.3)	3 (100)	0 (0.0)	0 (0.0)	0 (0.0)	0 (0.0)

*S*. *aureus* (*N* = 3)	S	—	1 (33.3)	2 (66.7)	0 (0.0)	2 (66.7)	3 (100)	3 (100)	3 (100)
	I	—	0 (0.0)	0 (0.0)	1 (33.3)	0 (0.0)	0 (0.0)	0 (0.0)	0 (0.0)
	R	—	2 (66.7)	1 (33.3)	2 (66.7)	1 (33.3)	0 (0.0)	0 (0.0)	0 (0.0)

*Enterobacter* spp. (*N* = 3)	S	—	1 (33.3)	2 (66.7)	0 (0.0)	3 (100)	3 (100)	3 (100)	3 (100)
	I	—	0 (0.0)	1 (33.3)	1 (33.3)	0 (0.0)	0 (0.0)	0 (0.0)	0 (0.0)
	R	—	2 (66.7)	0 (0.0)	2 (66.7)	0 (0.0)	0 (0.0)	0 (0.0)	0 (0.0)

*Citrobacter* spp. (*N* = 2)	S	—	0	1 (50)	0 (0.0)	1 (50)	2 (100)	2 (100)	2 (100)
	I	—	2 (100)	1 (50)	0 (0.0)	1 (50)	0 (0.0)	0 (0.0)	0 (0.0)
	R	—	0 (0.0)	0 (0.0)	2 (100)	0 (0.0)	0 (0.0)	0 (0.0)	0 (0.0)

*E*. *faecalis* (*N* = 1)	S	—	0 (0.0)	0 (0.0)	0 (0.0)	1 (100)	1 (100)	1 (100)	1 (100)
	I	—	0 (0.0)	1 (100)	0 (0.0)	0 (0.0)	0 (0.0)	0 (0.0)	0 (0.0)
	R	—	1 (100)	0 (0.0)	1 (100)	0 (0.0)	0 (0.0)	0 (0.0)	0 (0.0)

*Streptococcus* spp. (*N* = 0)	S	—	0 (0.0)	0 (0.0)	0 (0.0)	0 (0.0)	0 (0.0)	0 (0.0)	0 (0.0)
	I	—	0 (0.0)	0 (0.0)	0 (0.0)	0 (0.0)	0 (0.0)	0 (0.0)	0 (0.0)
	R	—	0 (0.0)	0 (0.0)	0 (0.0)	0 (0.0)	0 (0.0)	0 (0.0)	0 (0.0)

Total, *n* (%)	S	—	12 (25.5)	21 (44.7)	1 (2.2)	39 (83.0)	46 (97.9)	45 (95.8)	45 (95.8)
	I	—	5 (10.6)	5 (10.6)	5 (10.6)	3 (6.4)	1 (2.1)	2 (4.2)	1 (2.1)
	R	—	30 (63.9)	21 (44.7)	41 (87.2)	5 (10.6)	0 (0.0)	0 (0.0)	1 (2.1)

S: number of sensitive; I: number of intermediate; R: number of resistant isolates; -antibiotic was not used.

**Table 3:** Antimicrobial susceptibility pattern of uropathogens isolated from urine specimens of outpatients.

Bacterial isolates	Pattern	Antimicrobial susceptibility pattern, *n* (%)							
		Amoxicillin/C	Cephalothin	Ceftriaxone	Tetracycline	Gentamicin	Ciprofloxacin	Nitrofurantoin	Norfloxacin
*E*. *coli* (*N* = 11)	S	2 (18.2)	2 (18.2)	2 (18.2)	0 (0.0)	9 (81.8)	10 (90.9)	11 (100)	11 (100)
	I	3 (27.3)	3 (27.3)	5 (45.4)	2 (18.2)	2 (18.2)	1 (9.1)	0 (0.0)	0 (0.0)
	R	6 (54.5)	6 (54.5)	4 (36.4)	9 (81.8)	0 (0.0)	0 (0.0)	0 (0.0)	0 (0.0)

*Klebsiella* spp. (*N* = 3)	S	0 (0.0)	1 (33.3)	2 (66.7)	0 (0.0)	2 (66.7)	3 (100)	3 (100)	3 (100)
	I	1 (33.3)	0 (0.0)	1 (33.3)	1 (33.3)	1 (33.3)	0 (0.0)	0 (0.0)	0 (0.0)
	R	2 (66.7)	2 (66.7)	0 (0.0)	2 (66.7)	0 (0.0)	0 (0.0)	0 (0.0)	0 (0.0)

*Proteus* spp. (*N* = 3)	S	0 (0.0)	2 (66.7)	2 (66.7)	0 (0.0)	2 (66.7)	3 (100)	3 (100)	3 (100)
	I	0 (0.0)	0 (0.0)	0 (0.0)	1 (33.3)	1 (33.3)	0 (0.0)	0 (0.0)	0 (0.0)
	R	3 (100)	1 (33.3)	1 (33.3)	2 (66.7)	0 (0.0)	0 (0.0)	0 (0.0)	0 (0.0)

*Pseudomonas* spp. (*N* = 3)	S	—	0 (0.0)	1 (33.3)	0 (0.0)	2 (66.7)	3 (100)	3 (100)	2 (66.7)
	I	—	1 (33.3)	0 (0.0)	0 (0.0)	0 (0.0)	0 (0.0)	0 (0.0)	0 (0.0)
	R	—	2 (66.7)	2 (66.7)	3 (100)	1 (33.3)	0 (0.0)	0 (0.0)	1 (33.3)

CNS (*N* = 2)	S	1 (50)	1 (50)	1 (50)	0 (0.0)	2 (100)	2 (100)	2 (100)	2 (100)
	I	0 (0.0)	1 (50)	0 (0.0)	1 (50)	0 (0.0)	0 (0.0)	0 (0.0)	0 (0.0)
	R	1 (50)	0 (0.0)	1 (50)	1 (50)	0 (0.0)	0 (0.0)	0 (0.0)	0 (0.0)

*S*. *aureus* (*N* = 2)	S	1 (50)	0 (0.0)	2 (100)	0 (0.0)	2 (100)	2 (100)	2 (100)	2 (100)
	I	0 (0.0)	1 (50)	0 (0.0)	1 (50)	0 (0.0)	0 (0.0)	0 (0.0)	0 (0.0)
	R	1 (50)	1 (50)	0 (0.0)	1 (50)	0 (0.0)	0 (0.0)	0 (0.0)	0 (0.0)

*Enterobacter* spp. (*N* = 2)	S	0 (0.0)	1 (50)	2 (100)	0 (0.0)	2 (100)	2 (100)	2 (100)	2 (100)
	I	1 (50)	1 (50)	0 (0.0)	0 (0.0)	0 (0.0)	0 (0.0)	0 (0.0)	0 (0.0)
	R	1 (50)	0 (0.0)	0 (0.0)	2 (100)	0 (0.0)	0 (0.0)	0 (0.0)	0 (0.0)

*Citrobacter* spp. (*N* = 1)	S	0 (0.0)	0 (0.0)	1 (100)	0 (0.0)	1 (100)	1 (100)	1 (100)	1 (100)
	I	0 (0.0)	1 (100)	0 (0.0)	0 (0.0)	0 (0.0)	0 (0.0)	0 (0.0)	0 (0.0)
	R	1 (100)	0 (0.0)	0 (0.0)	1 (100)	0 (0.0)	0 (0.0)	0 (0.0)	0 (0.0)

*E*. *faecalis* (*N* = 0)	S	0 (0.0)	0 (0.0)	0 (0.0)	0 (0.0)	0 (0.0)	0 (0.0)	0 (0.0)	0 (0.0)
	I	0 (0.0)	0 (0.0)	0 (0.0)	0 (0.0)	0 (0.0)	0 (0.0)	0 (0.0)	0 (0.0)
	R	0 (0.0)	0 (0.0)	0 (0.0)	0 (0.0)	0 (0.0)	0 (0.0)	0 (0.0)	0 (0.0)

*Streptococcus* spp. (*N* = 1)	S	0 (0.0)	0 (0.0)	0 (0.0)	0 (0.0)	1 (100)	1 (100)	1 (100)	1 (100)
	I	0 (0.0)	0 (0.0)	0 (0.0)	0 (0.0)	0 (0.0)	0 (0.0)	0 (0.0)	0 (0.0)
	R	1 (100)	1 (100)	1 (100)	1 (100)	0 (0.0)	0 (0.0)	0 (0.0)	0 (0.0)

Total, *n* (%)	S	4 (16)	7 (25.0)	13 (46.4)	0 (0.0)	23 (82.1)	27 (96.4)	28 (100)	27 (96.4)
	I	5 (20)	8 (28.6)	6 (21.5)	6 (21.4)	4 (14.3)	1 (3.6)	0 (0.0)	0 (0.0)
	R	16 (64)	13 (46.4)	9 (32.1)	22 (78.6)	1 (3.6)	0 (0.0)	0 (0.0)	1 (3.6)

S: number of sensitive; I: number of intermediate; R: number of resistant isolates; -antibiotic was not used.

**Table 4:** Multiple antimicrobial resistance indexes of uropathogens isolated from inpatients.

Isolates	*A*	*B*	MARI = (a/*b*)	Antibiotics to which the isolates are resistant
*E*. *coli*	4	7	0.571	CEPH, CRO, TE, and CN
*Klebsiella* spp.	3	7	0.428	CEPH, CRO, and TE
*Proteus* spp.	4	7	0.571	CEPH, CRO, TE, and CN
*Pseudomonas* spp.	5	7	0.714	CEPH, CRO, TE, CN, and NOR
CNS	3	7	0.428	CEPH, CRO, and TE
*S*. *aureus*	4	7	0.571	CEPH, CRO, TE, and CN
*Enterobacter* spp.	2	7	0.285	CEPH, and TE
*Citrobacter* spp.	1	7	0.142	TE
*E*. *faecalis*	2	7	0.285	CEPH, and TE
*Streptococcus* spp.	—	—	—	—

Total number of antibiotics tested = 7; CEPH : cephalexin; CRO : ceftriaxone; TE : tetracycline; CN : gentamicin; CIP : ciprofloxacin; F : nitrofurantoin; NOR : norfloxacin.

**Table 5:** Multiple antimicrobial resistance indexes of uropathogens isolated from outpatients.

Isolates	*A*	*B*	MARI = (a/*b*)	Antibiotics to which the isolates are resistant
*E*. *coli*	4	8	0.500	AMC, CEPH, CRO, and TE
*Klebsiella* spp.	3	8	0.375	AMC, CEPH, and TE
*Proteus* spp.	4	8	0.500	AMC, CEPH, CRO, and TE
*Pseudomonas* spp.	5	8	0.625	CEPH, CRO, TE, CN, and NOR
CNS	3	8	0.375	AMC, CRO, and TE
*S*. *aureus*	3	8	0.375	AMC, CEPH, and TE
*Enterobacter* spp.	2	8	0.250	AMC, and TE
*Citrobacter* spp.	2	8	0.250	AMC, and TE
*E*. *faecalis*	—	—	—	—
*Streptococcus* spp.	4	8	0.500	AMC, CEPH, CRO, and TE

Total number of antibiotics tested = 8; AMC: amoxicillin/clavulanic acid; CEPH : cephalexin; CRO : ceftriaxone; TE : tetracycline; CN : gentamicin; CIP : ciprofloxacin; NFN : nitrofurantoin; NOR : norfloxacin.

## Data Availability

The data used to support the findings of this study are included within this article.

## Abbreviations

CLSI: Clinical Laboratory Standards Institute
CNS: Coagulase Negative Staphylococci
MARI: Multiple Antimicrobial Resistance Index
MHA: Muller-Hinton Agar
PGH: Pawe General Hospital
UTIs: Urinary Tract Infections.
